# SnAg_2_O_3_-Coated Adhesive Tape as a Recyclable Catalyst for Efficient Reduction of Methyl Orange

**DOI:** 10.3390/ma16216978

**Published:** 2023-10-31

**Authors:** Kalsoom Akhtar, Asma A. Alhaj, Esraa M. Bakhsh, Sher Bahadar Khan, Taghreed M. Fagieh

**Affiliations:** Chemistry Department, Faculty of Science, King Abdulaziz University, P.O. Box 80203, Jeddah 21589, Saudi Arabia; aalhaj0007@stu.kau.edu.sa (A.A.A.); ibakhsh@kau.edu.sa (E.M.B.); sbhkhan@kau.edu.sa (S.B.K.); tfagieh@kau.edu.sa (T.M.F.)

**Keywords:** SnAg_2_O_3_, SnAg_2_O_3_@AT, nanocatalyst, pollutants, methyl orange, reduction

## Abstract

Silver oxide-doped tin oxide (SnAg_2_O_3_) nanoparticles were synthesized and different spectroscopic techniques were used to structurally identify SnAg_2_O_3_ nanoparticles. The reduction of 4-nitrophenol (4-NP), congo red (CR), methylene blue (MB), and methyl orange (MO) was studied using SnAg_2_O_3_ as a catalyst. Only 1.0 min was required to reduce 95% MO; thus, SnAg_2_O_3_ was found to be effective with a rate constant of 3.0412 min^−1^. Being a powder, SnAg_2_O_3_ is difficult to recover and recycle multiple times. For this reason, SnAg_2_O_3_ was coated on adhesive tape (AT) to make it recyclable for large-scale usage. SnAg_2_O_3_@AT catalyst was assessed toward MO reduction under various conditions. The amount of SnAg_2_O_3_@AT, NaBH_4_, and MO was optimized for best possible reduction conditions. The catalyst had a positive effect since it speed up the reduction of MO by adding more SnAg_2_O_3_@AT and NaBH_4_ as well as lowering the MO concentration. SnAg_2_O_3_@AT totally reduced MO (98%) in 3.0 min with a rate constant of 1.3669 min^−1^. These findings confirmed that SnAg_2_O_3_@AT is an effective and useful catalyst for MO reduction that can even be utilized on a large scale for industrial purposes.

## 1. Introduction

Recently, much attention has been given to heterogeneous catalysis because of the enormously important role it plays in environmentally and industrially significant catalytic reactions. Catalysis has extensive applications, comprising the synthesis of crucial pharmaceutical scaffolds, the transformation of harmful chemicals into useful ones, the degradation and reduction of contaminants and toxins, etc. However, the effectiveness and durability of catalysts play a major part in catalysis [1,2,3]. Therefore, the primary goal of catalysis is to prepare stable and effective catalysts, which have high catalytic activities on a large scale. The catalyst’s ability to catalyze reactions is mostly determined by the catalyst’s active sites, the molecules’ ability to interact with the catalyst, and the mass transformation of the reacting molecules into the product [4,5,6]. Therefore, it is essential to design effective catalysts with extraordinary efficiency for industrial and environmental purposes [7,8,9].

The most effective catalysts are metal oxides, which are frequently utilized in numerous industrial and environmental applications [10,11,12,13,14,15]. Tin oxide (SnO_2_) nanomaterials have attracted a lot of attention and have proven their efficiency as crucial heterogeneous catalysts. SnO_2_ is an effective catalyst that has been used in a variety of environmental applications and synthetic reactions. SnO_2_ has been employed in photo-catalysis, catalysis, Li-ion batteries, sensing, and other processes [16,17,18]. However, doped metal oxides and metal oxide nanocomposites are substantially more effective, stable, and competent catalysts for several processes, therefore, it has gained considerable attention. Metal oxides can be doped with other metals to increase their surface area and reaction sites, which improves the catalyst’s ability to catalyze various reactions. Because doping affects the characteristics of metal oxides and demonstrated outstanding catalytic activity in several catalytic reactions [19,20,21,22]. Thus, in response to the growing demand for synthetic and environmental applications, silver oxide-tin oxide nanocomposite was prepared to improve catalytic efficiency. However, SnAg_2_O_3_ is a powder, thus, one of the main issues with powder catalysts is that they tend to aggregate, which limits its ability to catalyze. The powder catalyst also cannot be recycled and used again by merely recovering it from the reaction medium. Therefore, to make the catalyst easier to reuse, polymeric or other supports can be employed since they play a significant role as hosts for metal oxides and other nanomaterials [23,24,25,26]. Benali et al. developed a catalyst based on a Zn(II)-crosslinked alginate loaded with Ag NPs for dye reduction [27]. Mallakpour et al. reported the catalytic reduction of nitrophenols, dyes, and metal ion pollutants based on mesoporous Ca-alginate/melamine-rich covalent organic polymer/cupric oxide microgel beads [28]. Meena and Saini described the synthesis of a PANI/ZnO/MnO_2_ ternary nanocomposite which was employed in the catalytic reduction of 4-nitrophenol as well as the adsorption of crystal violet dye from aqueous solutions [29].

Here, SnAg_2_O_3_ nanoparticles were synthesized and structurally characterized via numerous techniques. After that, the catalytic performance of SnAg_2_O_3_ was evaluated for the catalytic reduction of several contaminants. To examine the catalytic ability of SnAg_2_O_3_, the catalytic reduction of dyes and nitrophenols was used as a model electron exchange process. SnAg_2_O_3_ was discovered to be an effective catalyst and subsequently used for catalytic reactions involving electron transfers in 4-NP, MO, CR, and MB reduction. SnAg_2_O_3_ was found to be effective for MO reduction and it was revealed that the catalyst amount has a beneficial impact and greatly improves the reaction rate. Further, SnAg_2_O_3_ was coated on AT to make the catalyst easier to be recycled. In the reduction of MO, BH_4_^−^ ions diffuse into the surface of SnAg_2_O_3_ and SnAg_2_O_3_@AT in the first stage, where borohydride injects electrons onto the catalyst. Then, the electrons are subsequently transferred to MO. These catalysts serve as an electron relay. Thus, SnAg_2_O_3_ and SnAg_2_O_3_@AT are excellent nanocatalysts for the reduction of MO. The prepared nanocatalysts have the potential to compete and eventually substitute the well-known commercial catalysts because of their superior efficiency, cost-effectiveness, and simplicity in processing. The novelty of the developed efficient catalyst is that it overcomed the limitations like aggregation, regeneration, and non-recyclability. These limitations could be avoided by coating SnAg_2_O_3_ on AT support which made the reuse of the SnAg_2_O_3_ catalyst easier. AT was used as a support on which SnAg_2_O_3_ was coated by just rubbing where the glue had bound the catalyst. The coating of a powder catalyst is an ideal method for the advancement of the catalyst because it is a challenge to implement powder nanomaterials as a catalyst for large scale application. The coating of powder nanomaterials on a flexible support was developed to address the problems of aggregation and recyclability. This allowed powder catalysts to be recyclable and widen the catalyst applicability. 

## 2. Experimental 

### 2.1. Reagents and Chemicals

Sodium borohydride (NaBH_4_, 97%) was purchased from BDH chemicals, England. Silver chloride, tin chloride, 4-nitrophenol (4-NP), congo red (CR), methyl orange (MO), and methylene blue (MB) were purchased from Sigma-Aldrich. To prepare all solutions, distilled water was utilized. Adhesive tape, which is normally used for packing purposes, was bought from the local market and used as a supporting glue-based tape for the catalyst.

### 2.2. Preparation of SnAg_2_O_3_ and SnAg_2_O_3_@AT

**Scheme 1:**
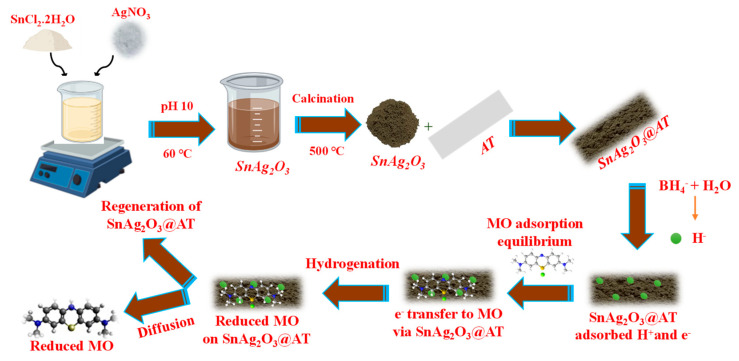
Graphic illustration of SnAg_2_O_3_@AT nanocatalyst preparation and its application in MO reduction.

Initially, the solution was made by placing the salts of AgNO_3_ and SnCl_2_.2H_2_O in 100 mL of water, and stirring the mixture (1:1 molar ratio) for 30 min. Then, the pH of the solution was adjusted to 10 by dropping NaOH solution, and the solution was stirred continuously at 60.0 °C overnight while being kept on a hot plate stirrer [17,18]. As soon as the reaction was stopped, SnAg_2_O_3_ settled down as a precipitate. Then, SnAg_2_O_3_ was washed, dried, and then further calcined for five hours at 500 °C. SnAg_2_O_3_@AT was prepared by coating SnAg_2_O_3_ on AT, as shown in Scheme 1.

### 2.3. Characterization of SnAg_2_O_3_

The morphology of SnAg_2_O_3_ was examined using a JEOL Scanning Electron Microscope (JSM-7600F, Akishima-shi, Japan). SnAg_2_O_3_ elemental composition was determined using the Oxford-EDS technique for Energy-Dispersive X-Ray Spectrometry (EDS). Thermo Scientific’s (Waltham, MA, USA) X-ray diffractometer was used to study the crystal structure of SnAg_2_O_3_. Thermo Fisher Scientific’s NicoletTM iS50 FTIR Spectrometer was used to identify the functional groups of SnAg_2_O_3_. Using a Thermo Scientific Evolution 300 UV-vis spectrometer, spectral data for all chemicals were recorded over the wavelength range of 200–800 nm.

### 2.4. Catalytic Studies of SnAg_2_O_3_ and SnAg_2_O_3_@AT

SnAg_2_O_3_ was initially used for the selectivity study of a reduction reaction of 4-NP, CR, MB, and MO. Each compound’s reduction was evaluated after 2.5 mL of 4-NP (0.1 mM), CR (0.07 mM), MB (0.07 mM), and MO (0.07 mM) were individually mixed with 0.5 mL of NaBH_4_ (0.1 M) and verified each compound’s decrease. The reduction was then observed by continuously measuring the UV-vis spectra of CR, MB, and MO at maximum wavelengths of 488 nm, 660 nm, and 460 nm, respectively. However, after adding NaBH_4_, 4-NP was tested at 400 nm due to the production of nitrophenolate ions. Next, SnAg_2_O_3_ (20 mg) was added to each compound. Because SnAg_2_O_3_ was found to be more efficient and selective for MO reduction, SnAg_2_O_3_@AT was evaluated for further reduction studies using 0.02 mM, 0.05 mM, and 0.07 mM of MO, with 0.3 mL, 0.5 mL, and 1.0 mL of NaBH_4_, using 5.5, 8.5 and 9.5 mg of SnAg_2_O_3_@AT, and different reduction parameters were optimized.

The following Equation (1) was used to estimate the percent reduction of each pollutant [30,31,32]:

(1) $$\%Reduction=\frac{C_{o}-C_{t}}{C_{o}}\times 100=\frac{A_{o}-A_{t}}{A_{o}}\times 100$$

where *C_o_* = *A_o_* = initial concentration and *C_t_* = *A_t_* = final concentration of each compound.

## 3. Results and Discussion

### 3.1. Catalyst Selection

The efficiency, stability, and reusability of the catalyst are three crucial considerations that should be taken into account in preparing an effective catalyst. Several catalysts are effective; however, they have issues with stability and reusability. In order to minimize the recyclability difficulties and provide a reliable catalyst for industrial and commercial large-scale use, an effective catalyst was prepared based on SnAg_2_O_3_ and further used to coat an AT. SnAg_2_O_3_ was made as a powder, however, powder catalysts typically suffer from aggregation, separation, and recyclability issues. Several spectroscopy approaches were used to structurally study the prepared SnAg_2_O_3_ and SnAg_2_O_3_@AT.

### 3.2. Physiochemical Characterization of SnAg_2_O_3_

**Figure 1:**
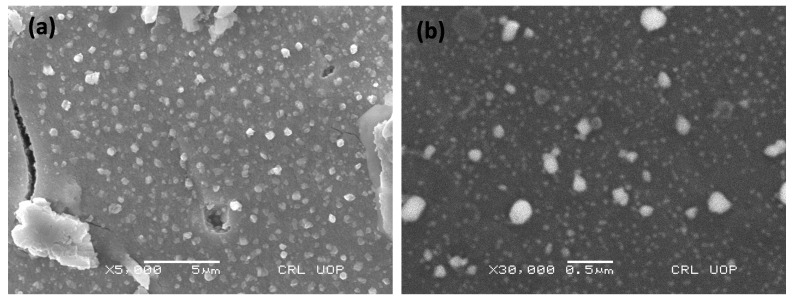
SEM images of the SnAg_2_O_3_ nanocatalyst at (**a**) low and (**b**) high magnifications.

SEM images of the SnAg_2_O_3_ nanocatalyst, shown in Figure 1, were used to determine its size and surface morphology. According to the SEM pictures at various magnifications, SnAg_2_O_3_ had a morphology like well-scattered particles. The particles were found to have grown in significant numbers while maintaining their spherical form.

**Figure 2:**
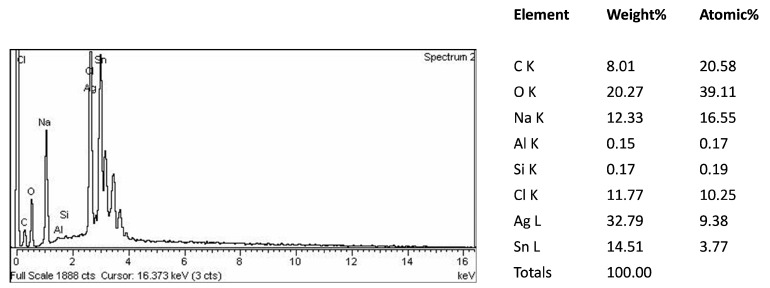
EDS spectrum and weight percent of SnAg_2_O_3_ nanocatalyst.

The elemental composition of SnAg_2_O_3_ was verified via EDS. According to the EDS analysis, Sn, Ag, and O peaks were observed at around 1.5, 2.6 and 3.0 kV with respective weight percentages of 14.51%, 32.79%, and 20.27%, respectively, as shown in Figure 2. The spectrum showed that SnAg_2_O_3_ comprised Sn, Ag, and O, in which Ag had the highest mass percentage, indicating that it was the primary component of SnAg_2_O_3_. The EDS spectrum indicated that the preparation of SnAg_2_O_3_ was successful.

**Figure 3:**
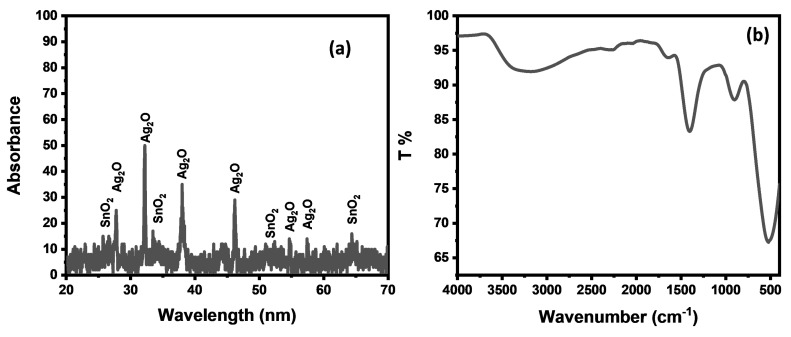
(**a**) XRD and (**b**) ATR-FTIR of SnAg_2_O_3_ nanocatalyst.

X-ray diffraction confirmed the crystal structure for SnAg_2_O_3_ (Figure 3a). The SnAg_2_O_3_ XRD pattern showed many intense peaks at 26.6°, 27.8°, 32.2°, 33.5°, 38.0°, 46.1°, 52.2°, 54.7°, 57.4°, and 64.4°. The typical diffraction peaks of low intensities at 26.6°, 33.5°, 52.2°, and 64.4°, which were indexed to (110), (101), (211), and (301) planes fitting with tetragonal SnO_2_. The characteristic hexagonal Ag_2_O peaks of (110), (111), (200), (211), (220), and (221) planes were shown at 27.8°, 32.2°, 38.0°, 46.1°, 54.4°, and 57.4°, respectively. The XRD pattern perfectly matched the hexagonal Ag_2_O and tetragonal SnO_2_ crystal structures [33,34]. This indicated that the material includes both Ag_2_O and SnO_2_ according to the XRD spectrum of SnAg_2_O_3_. 

ATR-FTIR analysis was used to evaluate the SnAg_2_O_3_ nanocatalyst chemical composition. The SnAg_2_O_3_ ATR-FTIR spectrum showed a strong band at 519 cm^−1^ (Figure 3b) that was responsible for the M–O bond (M = Sn, Ag). Additionally, the OH group broad bands were observed at 3200 cm^−1^ and 1637 cm^−1^. It also showed two bands at 901 cm^−1^ and 1404 cm^−1^, which were responsible for the nitrate group, suggesting that SnAg_2_O_3_ may contain the nitrate of the precursor. The FTIR spectrum of the prepared nanocatalyst supports the growth of SnAg_2_O_3_.

## 4. Catalytic Properties of SnAg_2_O_3_

**Figure 4:**
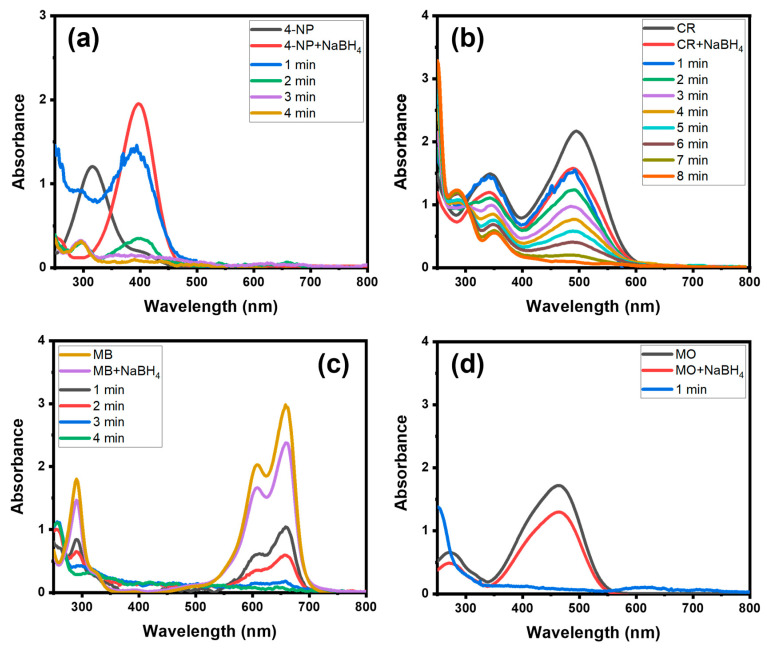
Time-dependent UV–vis spectra of (**a**) 4-NP, (**b**) CR, (**c**) MB, and (**d**) MO using 20 mg of SnAg_2_O_3_ and 0.5 mL of NaBH_4_.

**Figure 5:**
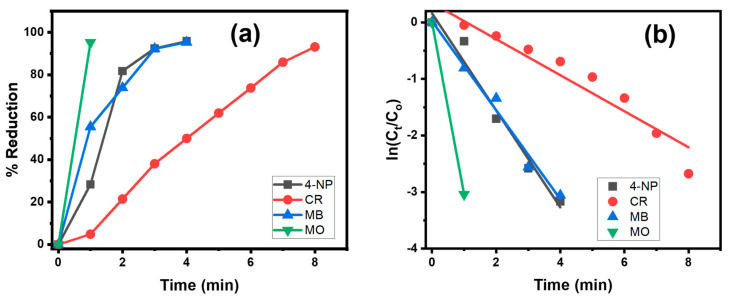
(**a**) % Reduction and (**b**) kinetics using 20 mg of SnAg_2_O_3_ and 0.5 mL of NaBH_4_ for reduction of different pollutants.

In order to assess the SnAg_2_O_3_ catalytic impact, it was first evaluated as a nanocatalyst for the reduction of 4-NP, CR, MB, and MO via a reducing agent. Initially, only a modest amount (20 mg) of SnAg_2_O_3_ was scrutinized in the reduction process of each individual pollutant. Figure 4 clearly shows that the absorbance of 4-NP, CR, MB, and MO gradually decreased, indicating that SnAg_2_O_3_ had a positive impact on their reduction. These findings confirm that the SnAg_2_O_3_ nanocatalyst reduced MO and CR within 1.0 min and 8.0 min, respectively, while it took 4.0 min to reduce both MB and 4-NP. Using Equation (1), the percentage reduction was further computed, and it was found that the SnAg_2_O_3_ nanocatalyst reduced 95.77% of 4-NP, 93.12% of CR, 95.23% of MB, and 95.22% of MO (Figure 5a).

Further, to examine the rate constants for all pollutants that are reduced in the presence of SnAg_2_O_3_, the pseudo-first-order kinetic presented in Equation (2) was used [35,36,37]:

ln C_t_/C_o_ = ln A_t_/A_o_ = −K_t_ (2)

where A_t_ = C_t_ is the absorbance or concentration of reduced pollutant at various reaction times, A_o_ = C_o_ is the absorbance or reducing chemical concentration prior to the reaction, and K (min^−1^) is the slope-based rate constant calculated from the graph of ln C_t_/C_o_ vs. SnAg_2_O_3_ reduction time (Figure 5b).

The rate constant values for 4-NP, CR, MB, and MO reduction reactions using SnAg_2_O_3_ were 0.8573 min^−1^, 0.3186 min^−1^, 0.7859 min^−1^, and 3.0412 min^−1^, respectively. This suggested that SnAg_2_O_3_ is selective for MO reduction. Therefore, SnAg_2_O_3_ was more efficient in reducing MO based on the rate constants that reflect its catalytic activity.

Since MO reduction was completed using SnAg_2_O_3_ in 1.0 min, it was selected for the comprehensive analysis and optimization of reaction conditions using the SnAg_2_O_3_@AT nanocatalyst. Only SnAg_2_O_3_@AT was used for further MO reduction investigations in order to minimize recycling concerns and provide a superior catalyst for large-scale applications since powder catalysts have problems with aggregation, separation, and recyclability.

### 4.1. Catalytic Activity of SnAg_2_O_3_@AT toward MO

SnAg_2_O_3_@AT was used for MO reduction to test the catalytic activity. The reduction of 2.5 mL of MO (0.07 mM) was first investigated in the presence of only NaBH_4_. Without SnAg_2_O_3_ or SnAg_2_O_3_@AT, a negligible reduction took place, and the concentration of MO was slightly changed. This demonstrates unequivocally that a catalyst is necessary for MO reduction. To reduce MO effectively, SnAg_2_O_3_@AT was added and the reaction was monitored by measuring UV-vis absorption. The NaBH_4_ + MO reaction was initially investigated with 8.5 mg of SnAg_2_O_3_@AT.

**Figure 6:**
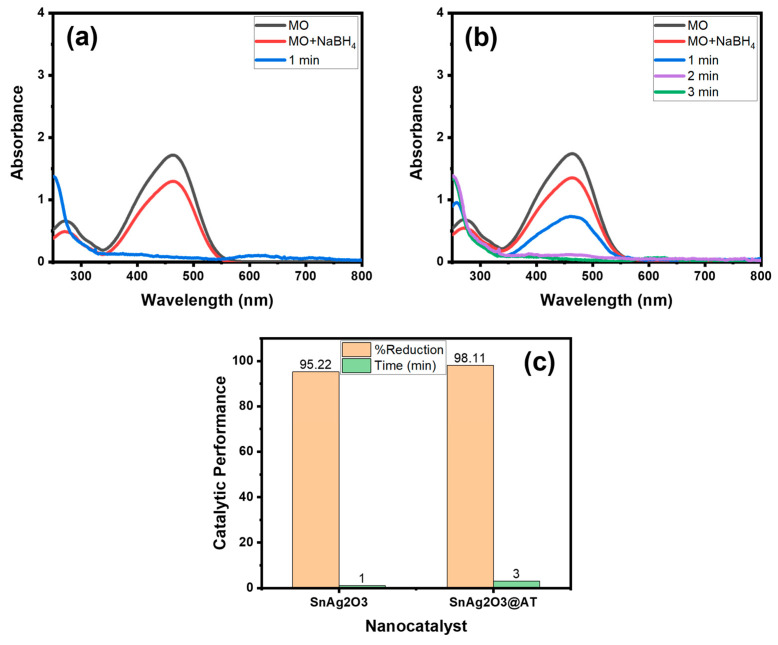
Time-dependent UV–vis spectra for MO reduction using (**a**) 20 mg of SnAg_2_O_3_ and (**b**) 8.5 mg of SnAg_2_O_3_@AT and 0.5 mL of NaBH_4_. (**c**) Comparison of nanocatalysts’ catalytic performance toward MO reduction.

Since there was a steady loss of the MO color as the reaction progressed along with the drop in absorbance, the reduction of MO was visually detected. This shift in color and drop in absorbance indicated MO reduction, which occurred in 1.0 min and 3.0 min by employing 20 mg of SnAg_2_O_3_ and 8.5 mg of SnAg_2_O_3_@AT, as shown in Figure 6.

**Figure 7:**
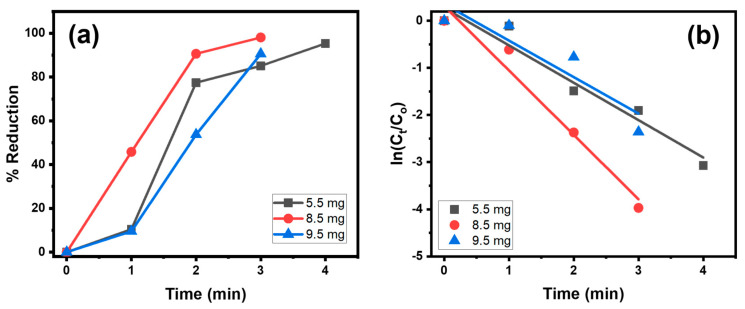
(**a**) % Reduction and (**b**) kinetics of MO reduction by 0.5 mL of NaBH_4_ using different amounts of SnAg_2_O_3_@AT (5.5 mg, 8.5 mg, and 9.5 mg).

Equation (2) was also used to determine the kinetic reduction of MO via SnAg_2_O_3_@AT. As a result, K was found to be 1.3669 min^−1^ (Figure 7b). As indicated in Table 1, the rate constants of SnAg_2_O_3_ and SnAg_2_O_3_@AT were also compared with those of other reported catalysts.

#### 4.1.1. Effect of SnAg_2_O_3_@AT Dosage

To explore the dose effect, 2.5 mL of MO was reduced by 0.5 mL of NaBH_4_ (0.1 M) in the presence of 5.5 mg, 8.5 mg, and 9.5 mg of SnAg_2_O_3_@AT. With a high dose of SnAg_2_O_3_@AT, the reduction process of MO occurred more rapidly. MO reduction with 8.5 mg and 9.5 mg of SnAg_2_O_3_@AT took only 3.0 min, which was faster than the lower dose (took 4.0 min). Equations (1) and (2) were used to determine the catalytic efficiency of SnAg_2_O_3_@AT in the reduction of MO, as illustrated in Figure 7, which showed that a superior reduction was quickly achieved by utilizing 8.5 mg of SnAg_2_O_3_@AT (98.11%) in just 3.0 min with a rate constant of 1.3669 min^−1^. By using 9.5 mg, SnAg_2_O_3_@AT reduced 90.56% of MO (K = 0.7751 min^−1^). On the other hand, 5.5 mg reduced 95.36% of MO in 4.0 min (K = 0.7936 min^−1^). These findings demonstrated that the amount of the catalyst has a beneficial effect on MO reduction because it is directly related to the percent reduction.

#### 4.1.2. Effect of NaBH_4_ Concentration

**Figure 8:**
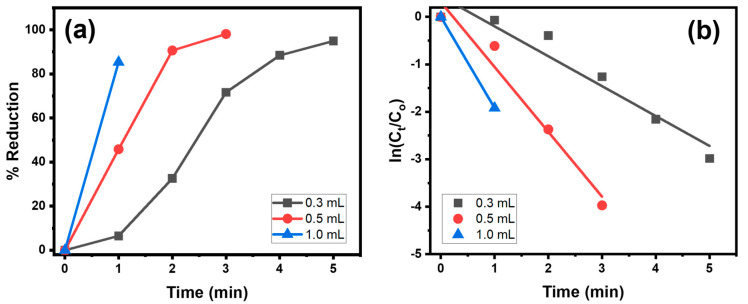
(**a**) % Reduction and (**b**) kinetics of MO reduction using 8.5 mg of SnAg_2_O_3_@AT in the presence of NaBH_4_ (0.3 mL, 0.5 mL, and 1.0 mL).

To examine the influence of NaBH_4_ concentration, 0.3 mL, 0.5 mL, and 1.0 mL of NaBH_4_ (1.0 M) were studied on the reduction of 2.5 mL of MO utilizing 8.5 mg of SnAg_2_O_3_@AT. The findings showed that NaBH_4_ had a negligible impact on SnAg_2_O_3_@AT ability to reduce MO. It was discovered that 8.5 mg of SnAg_2_O_3_@AT reduced 94.94% and 98.11% of MO in 5.0 min and 3.0 min, respectively, using 0.3 mL and 0.5 mL of NaBH_4_. While applying 1.0 mL of NaBH_4_, 8.5 mg of SnAg_2_O_3_@AT reduced 85.34% of MO in 1.0 min. As a result, it was discovered that by adding more NaBH_4_ (0.3 mL~1.0 mL), the MO reduction reaction rate was increased using SnAg_2_O_3_@AT, in which the rate constants were 0.6301 min^−1^, 1.3669 min^−1^, and 1.9199 min^−1^ for 0.3 mL, 0.5 mL, and 1.0 mL, respectively, as illustrated in Figure 8. Specifically, SnAg_2_O_3_@AT transfers electrons from BH_4_^−^ to MO. However, if the concentration of electron-rich moieties is in a large quantity, there is still competition for electrons to reach MO, which may reduce the % reduction of MO as a result of competition and steric hindrance. Overall, it can be concluded that the amount of NaBH_4_ has a good impact on MO reduction when it is used in appropriate amounts.

#### 4.1.3. Effect of MO Concentration

**Figure 9:**
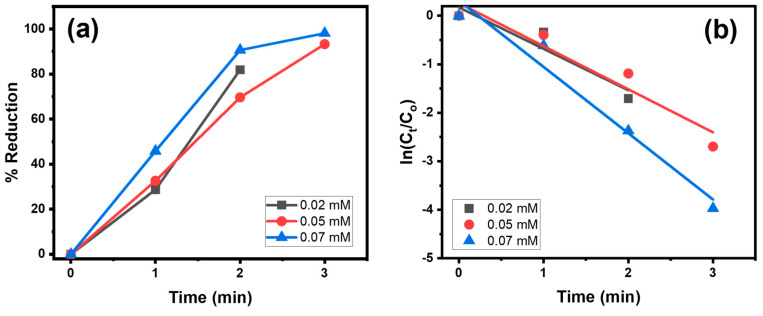
(**a**) % Reduction and (**b**) kinetics of MO reduction (0.02 mM, 0.05 mM, and 0.07 mM) using 8.5 mg of SnAg_2_O_3_@AT and 0.5 mL of NaBH_4_.

To assess the catalytic behavior of SnAg_2_O_3_@AT, the reduction process was tested using various concentrations of MO. A total of 8.5 mg of SnAg_2_O_3_@AT was utilized for the reduction of 2.5 mL of MO + 0.5 mL of NaBH_4_, in which the concentrations of MO were 0.02 mM, 0.05 mM, and 0.07 mM. As illustrated in Figure 9, it was discovered that SnAg_2_O_3_@AT reduced 0.02 mM MO much faster (2.0 min) than 0.05 mM and 0.07 mM (3.0 min). This means that higher concentrations of MO require more time to be reduced than lower concentrations. Accordingly, there is a direct proportion between the concentration of MO and its reduction time.

#### 4.1.4. Recyclability

**Figure 10:**
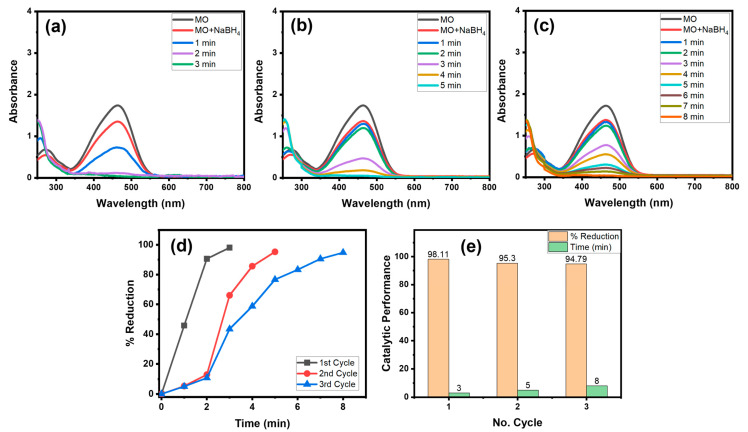
Time-dependent UV–vis spectra for reduction of 0.07 mM MO; (**a**) 1st cycle, (**b**) 2nd cycle, and (**c**) 3rd cycle using 8.5 mg of SnAg_2_O_3_@AT and 0.5 mL of NaBH_4_. (**d**) % Reduction of MO using 8.5 mg of SnAg_2_O_3_@AT for three cycles. (**e**) Recyclability performance of SnAg_2_O_3_@AT nanocatalyst in MO reduction.

The stability and reusability of a catalyst are important factors from an economic and environmental perspective [45,46,47]. Thus, SnAg_2_O_3_@AT was investigated for recyclability. The recyclability of SnAg_2_O_3_@AT was evaluated by performing a reduction of 2.5 mL MO (0.07 mM). Three further reactions were analyzed using 8.5 mg of SnAg_2_O_3_@AT. It was simple to remove SnAg_2_O_3_@AT from the reaction medium after each use. Then, SnAg_2_O_3_@AT was cleaned with distilled water before being used in the following process. In the first, second, and third cycles, the data showed that SnAg_2_O_3_@AT completely (>94%) reduced MO in 3.0, 5.0, and 8.0 min, respectively. Figure 10 shows the catalytic activity of SnAg_2_O_3_@AT in each cycle.

The results showed that SnAg_2_O_3_@AT was able to effectively reduce MO in 8.0 min up to the third cycle, which indicated the stability and recyclability of the material based on the catalytic activity in each cycle. These findings imply that SnAg_2_O_3_@AT can be used repeatedly without significant losing of its superior catalytic activity.

#### 4.1.5. Mechanism of MO Reduction

The process of the catalytic reduction of MO depends on catalyst-mediated electron transmittance. NaBH_4_ dissociates initially into BH_4_^−^ and gets adsorbed on SnAg_2_O_3_@AT, where it donates electrons to the surface of SnAg_2_O_3_@AT which then attack MO molecules. Azo bonds are activated by electrons existing on the surface of SnAg_2_O_3_@AT when MO molecules get adsorbed onto it. As a result, the breakage of azo bonds takes place, thus, the formation of aromatic amines occurs during the reduction process resulting in the disappearance of MO color which confirms the completion of its catalytic reduction.

## 5. Conclusions

This study demonstrated the straightforward manufacturing of SnAg_2_O_3_ nanomaterial, which was successfully produced using the co-precipitation method. With the help of SEM, EDS, XRD, and FTIR, the physiochemical characterization of SnAg_2_O_3_ was examined. The reduction of different compounds was investigated (4-NP, CR, MB, and MO) in the presence of SnAg_2_O_3_. Using SnAg_2_O_3_ and SnAg_2_O_3_@AT, MO was reduced within 1.0 min and 3.0 min with NaBH_4_ at reduction rates of 3.0412 min^−1^ and 1.3669 min^−1^, respectively. Thus, based on the catalytic activities, SnAg_2_O_3_ and SnAg_2_O_3_@AT were specified as efficient catalysts for the MO reduction. MO reduction was therefore selected for further analysis and reaction condition optimization. The results suggested that SnAg_2_O_3_@AT is a stable and recyclable catalyst for the catalytic reduction of industrial dyes.

## Figures and Tables

**Table 1:** Rate constant comparison of SnAg_2_O_3_ and SnAg_2_O_3_@AT with literature.

Catalyst	Rate Constant (min^−1^)	Reference
Alg-tin oxide–cobalt oxide	1.2775	[18]
AuNPs	0.1020	[38]
Pd NPs	0.0909	[39]
K-AgNPs	0.0286	[40]
WBs loaded with Ni NPs	0.1500	[41]
CuSnOS/AC	1.1480	[42]
Cu@Alg/BP	0.2702	[43]
Sd@Ag-NPs	0.3850	[44]
SnAg_2_O_3_	3.0412	This Study
SnAg_2_O_3_@AT	1.3669	This Study

## Data Availability

The data presented in this study are available on request from the corresponding author.
